# Sporadic Desmoid-Type Fibromatosis of the Small Intestine Muscularis Propria: A Case Report

**DOI:** 10.7759/cureus.84794

**Published:** 2025-05-25

**Authors:** Kai Nakao, Yusuke Okamura, Satoshi Kaihara, Azusa Uchimoto, Shigeo Hara

**Affiliations:** 1 Department of Surgery, Kobe City Medical Center General Hospital, Kobe, JPN; 2 Department of Pathology, Kobe City Medical Center General Hospital, Kobe, JPN

**Keywords:** desmoid tumor, familial adenomatous polyposis, jejunal neoplasm, laparotomy, mesenteric invasion, muscularis propria, small bowel tumor, spindle cell tumor, sporadic case, β-catenin immunostaining

## Abstract

Desmoid-type fibromatosis is a rare, locally aggressive tumor of fibroblastic origin that typically arises from the mesentery or abdominal wall. Sporadic desmoid-type fibromatosis originating from the small intestinal wall is extremely rare and presents unique diagnostic challenges due to its nonspecific imaging findings and lack of mucosal involvement.

A 69-year-old man with a history of prostate cancer and right renal cell carcinoma underwent routine postoperative surveillance, during which a proximal jejunal mass was incidentally detected on contrast-enhanced computed tomography (CT). Fluorodeoxyglucose-positron emission tomography/computed tomography (FDG-PET/CT) revealed moderate FDG uptake (maximum standardized uptake value (SUVmax), 3.62), raising suspicion for gastrointestinal stromal tumor or metastatic disease. Double-balloon enteroscopy showed no mucosal or submucosal lesions. Surgical exploration revealed four masses, including one arising from the muscularis propria of the jejunum and three within the mesentery. The jejunum was resected over a length of 80 cm, and reconstruction was performed using functional end-to-end anastomosis after extensive duodenal mobilization. Histopathological examination confirmed desmoid-type fibromatosis. The patient recovered uneventfully.

This case highlights the diagnostic difficulty and potential for multifocality in sporadic intra-abdominal desmoid-type fibromatosis. It underscores the importance of including desmoid-type fibromatosis in the differential diagnosis of mesenteric or submucosal tumors, especially in patients with a history of abdominal surgery. Surgical exploration and tailored reconstruction strategies are essential for achieving curative resection while preserving bowel length. Given the patient’s history of prior abdominal surgeries, this case supports the hypothesis that surgical trauma may contribute to tumor development in sporadic desmoid-type fibromatosis.

## Introduction

Desmoid tumors account for less than 0.3% of all soft tissue tumors [1], and primary tumors arising in the intestinal wall are extremely rare. Most previously reported intestinal desmoid tumors are associated with familial adenomatous polyposis (FAP) [2], while sporadic cases without FAP are scarce [3]. Due to its aggressive local behavior and high recurrence rate, appropriate diagnosis and treatment planning are critical [4]. Here, we present a case of a sporadic intestinal desmoid tumor and discuss the diagnostic challenges and surgical strategy, with reference to relevant case reports.

## Case presentation

**Figure 1:**
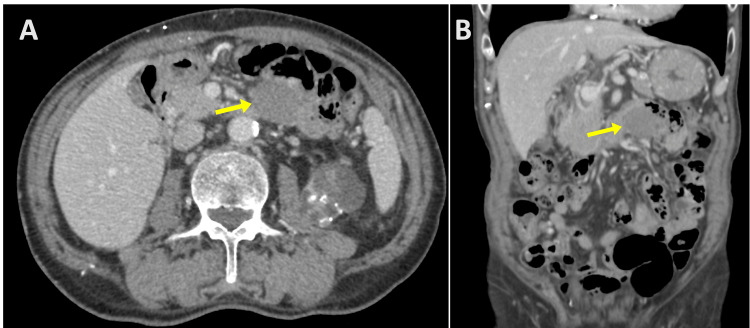
Contrast-enhanced CT images of the proximal jejunal tumor. (A) Axial and (B) coronal views reveal a well-circumscribed, homogenous mass measuring 36 × 30 × 35 mm located in the proximal jejunum. The mass exhibited mild and uniform enhancement, with no signs of invasion into adjacent organs.

In August 2024, a 69-year-old man with a history of prostate cancer and right renal cell carcinoma, both treated surgically, underwent routine postoperative surveillance 2.5 years after nephrectomy. Contrast-enhanced computed tomography (CT) revealed a well-circumscribed, mildly enhanced, homogeneous mass (36 × 30 × 35 mm) in the proximal jejunum (Figures 1A, 1B). He was asymptomatic; physical examination showed no abdominal tenderness or palpable mass. Laboratory tests showed no elevation of tumor markers, including carcinoembryonic antigen and carbohydrate antigen 19-9.

**Figure 2:**
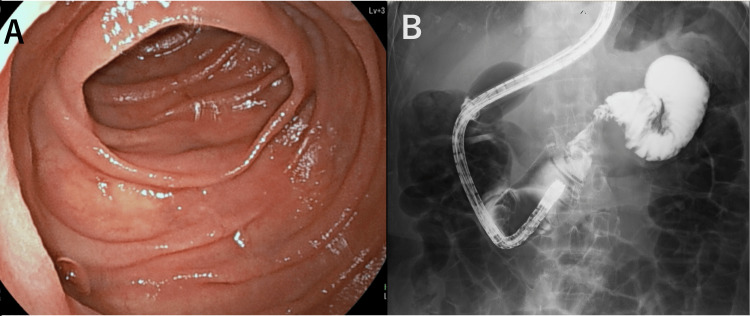
Double-balloon enteroscopy images. (A) Double-balloon enteroscopy revealing no mucosal lesions or submucosal masses in the proximal jejunum. (B) Contrast study showing no luminal narrowing or intraluminal protrusions, supporting the absence of endoluminal pathology.

**Figure 3:**
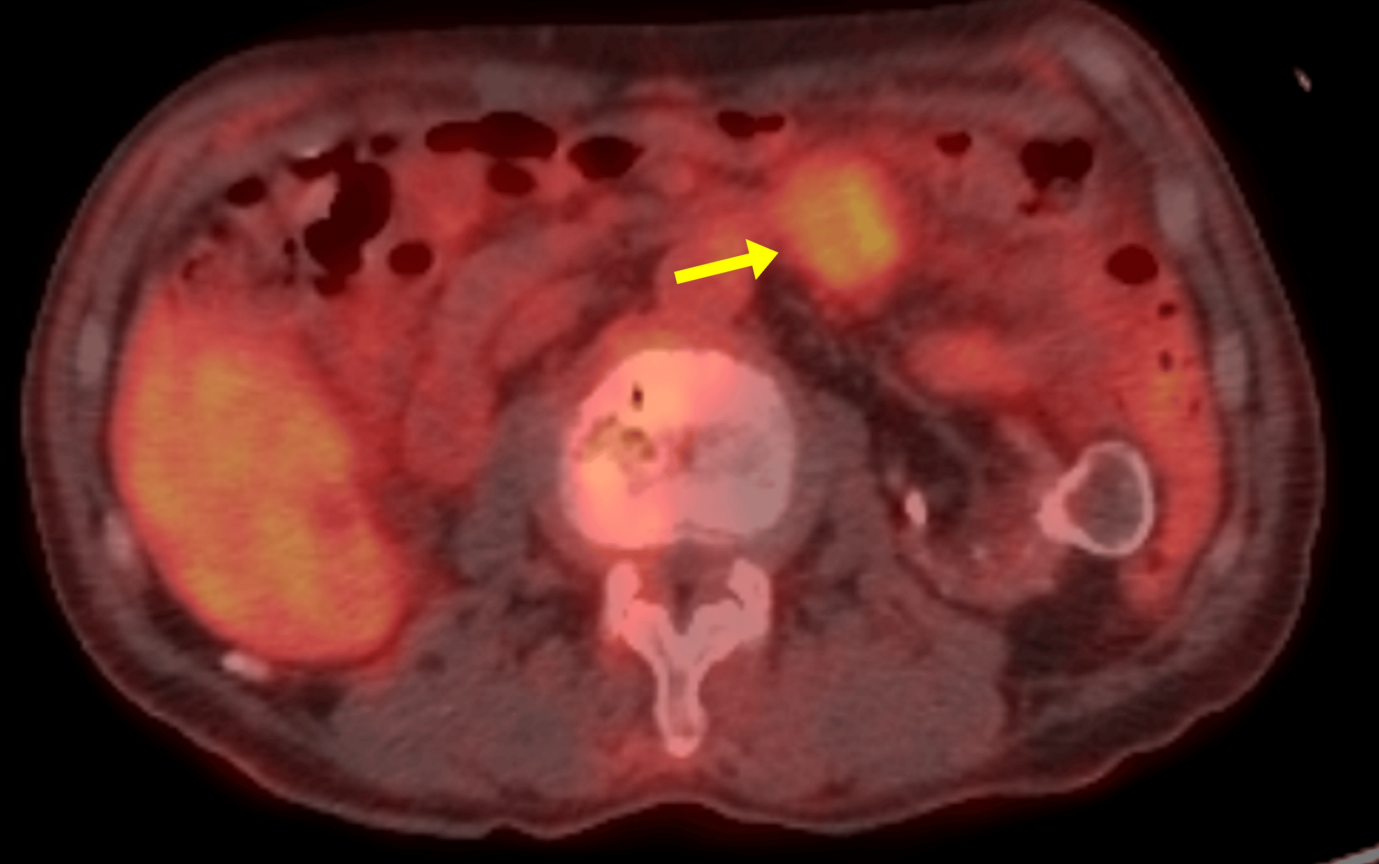
Fluorodeoxyglucose-positron emission tomography CT findings of the proximal jejunal tumor. Uptake was observed in the proximal jejunal mass, with a maximum standardized uptake value of 3.62.

Given the patient’s oncologic history, further evaluation was conducted to rule out malignancy or metastasis. Double-balloon enteroscopy revealed no mucosal or submucosal lesions in the proximal jejunum (Figure 2A), and no luminal narrowing or intraluminal protrusions were seen on contrast imaging (Figure 2B). Colonoscopy revealed no abnormalities. Fluorodeoxyglucose-positron emission tomography/computed tomography (FDG-PET/CT) demonstrated moderate uptake in the jejunal mass (maximum standardized uptake value (SUVmax), 3.62) (Figure 3).

Due to the diagnostic uncertainty and potential for malignancy, surgical resection was performed for definitive diagnosis and treatment.

Intraoperative findings and surgical procedure 

**Figure 4:**
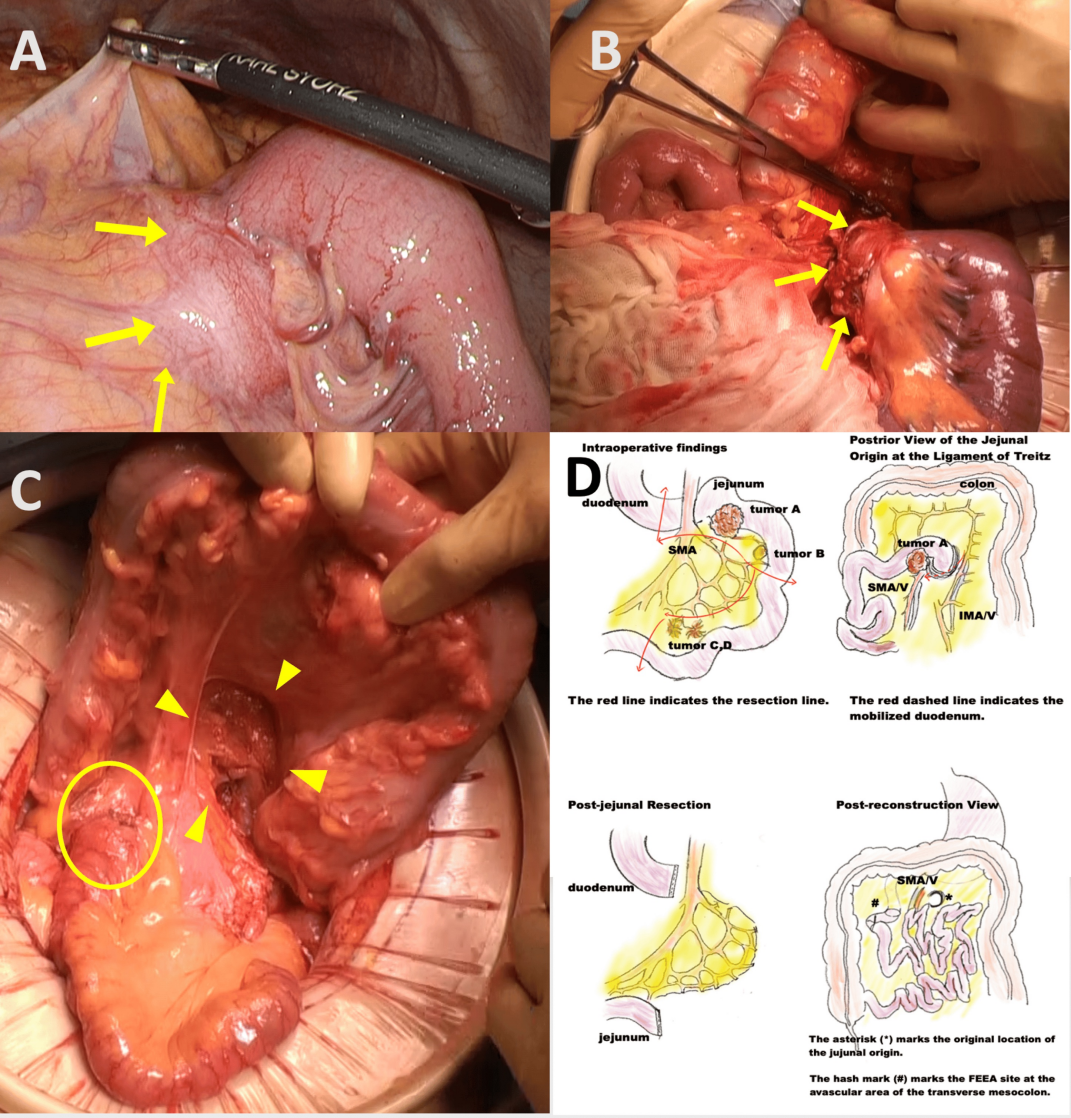
Intraoperative findings and surgical reconstruction following resection of a jejunal tumor. (A) Laparoscopic view showing a tumor at the jejunal origin with invasion into the mesentery. (B) Open surgical view after conversion from laparoscopy, directly visualizing the same proximal jejunal tumor. (C) Post-reconstruction view: the mobilized duodenum was passed through an avascular area of the transverse mesocolon to the right of the superior mesenteric artery, and a functional end-to-end anastomosis (FEEA) was created between the duodenum and jejunum. The anastomosis site is circled, and the arrowhead indicates the opened ligament of Treitz, corresponding to the original jejunal origin. (D) Surgical sketch showing duodenal mobilization and FEEA.

**Figure 5:**
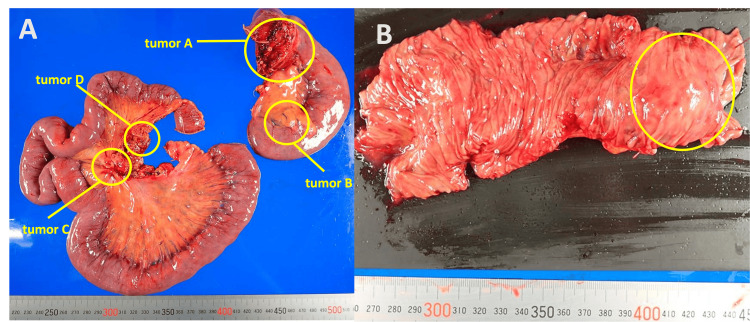
Resected specimen of the jejunal tumors. (A) Overview of the surgical specimen showing multiple nodular lesions in the proximal jejunum and mesenteric tissue. Tumor A: Tumor of jejunal origin with mesenteric invasion observed on preoperative computed tomography. Tumor B: Tumor found in the mesentery distal to tumor A intraoperatively. Tumors C and D: Tumors found in the mesentery of the jejunum during surgery. (B) Tumor A at the origin of the jejunum showing the morphology of a submucosal tumor.

Laparoscopy revealed tumor A at the origin of the jejunum with mesenteric invasion, necessitating conversion to open surgery (Figures 4A, 4B). Tumor B was found just distal to tumor A, and tumors C and D were identified approximately 60 cm further downstream in the jejunal mesentery. The jejunum, including all tumors, was resected over a length of 80 cm. To ensure sufficient proximal margin and allow safe reconstruction, the ligament of Treitz was transected, and the horizontal to descending duodenum was mobilized retroperitoneally. The duodenum was guided through an avascular area of the transverse mesocolon to the right of the superior mesenteric artery (SMA), and a functional end-to-end anastomosis (FEEA) was performed between the duodenum and jejunum (Figure 4C). The resected specimen clearly revealed multiple nodular lesions corresponding to the intraoperatively identified tumors A, B, C, and D. Tumor A, located at the jejunal origin, exhibited submucosal tumor-like features, while tumors B, C, and D were located within the jejunal mesentery. These findings are illustrated in Figure 5.

Pathological finding 

**Figure 6:**
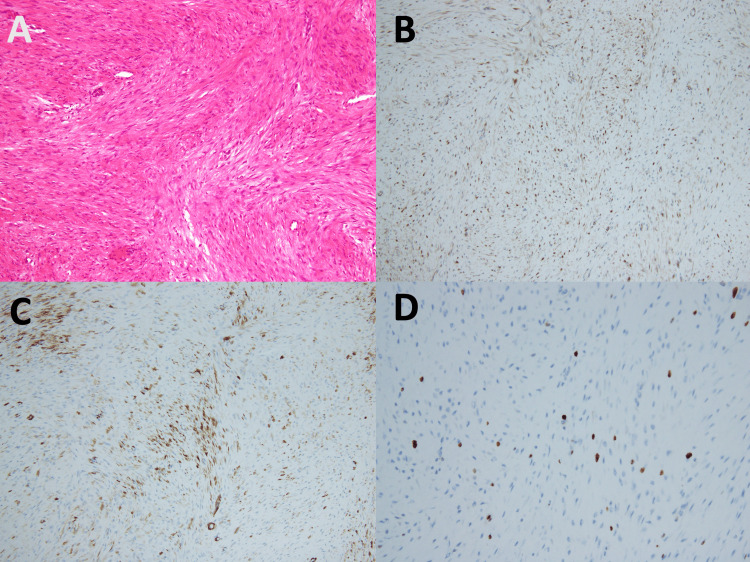
Histopathological and immunohistochemical findings of tumor A. (A) Hematoxylin and eosin staining revealing proliferation of bland spindle-shaped cells forming interlacing fascicles continuous with the muscularis propria. No mitotic figures are observed. (B) Immunohistochemistry showing focal nuclear and cytoplasmic positivity for β-catenin. (C) Partial positivity for α-SMA. (D) The Ki-67 labeling index was 2%.

Tumor A consisted of bland, spindle-shaped cells arranged in interlacing fascicles arising from the muscularis propria (Figure 6A). No mitotic figures were observed. Immunohistochemistry revealed focal nuclear and cytoplasmic positivity for β-catenin (Figure 6B), partial positivity for α-smooth muscle actin (SMA) (Figure 6C), and negativity for c-Kit, S-100, and CD34. The Ki-67 labeling index was 2% (Figure 6D). The diagnosis was confirmed to be desmoid-type fibromatosis. Tumors C and D showed no histological evidence of lymphoid structures and shared the same spindle-cell morphology and immunohistochemical profile as tumor A, confirming their identity as desmoid-type fibromatosis rather than lymph node involvement. All the resection margins were negative (R0 resection). Tumor B was diagnosed as a heterotopic pancreas unrelated to the desmoid tumor.

Postoperative course

The patient was discharged on postoperative day 16. At three months postoperatively, no recurrence was observed on contrast-enhanced CT.

## Discussion

Desmoid tumors are rare fibroblastic neoplasms with strong local invasiveness and a high recurrence rate, accounting for <0.3% of soft tissue tumors and <0.03% of all malignancies [1,3]. The present case involved a rare sporadic desmoid tumor originating from the small bowel wall. Differentiating solid tumors of the intestinal wall or mesentery can be challenging. Based on preoperative imaging, differential diagnoses included exophytic gastrointestinal stromal tumor, metastatic lesions from renal or prostate cancer, and lymphoma. Although desmoid-type fibromatosis is often associated with FAP [2], this patient showed no colorectal polyps or abnormalities on colonoscopy, making FAP unlikely.

Sporadic desmoid tumors, in contrast to FAP-associated cases, may arise following surgical trauma or chronic mechanical stress [5,6]. The present case had a history of multiple abdominal and pelvic surgeries, including nephrectomy and prostatectomy. These previous surgical interventions may have contributed to tumorigenesis. Moreover, sporadic desmoid tumors are often associated with mutations in the CTNNB1 gene, encoding β-catenin [7]. Although genetic testing was not performed in this case, immunohistochemistry revealed focal nuclear and cytoplasmic positivity for β-catenin, supporting the diagnosis.

Intraoperatively, multiple lesions not detected preoperatively were identified, which may have been missed due to the lack of mucosal involvement and nonspecific imaging features. This highlights the importance of meticulous intraoperative palpation and exploration in suspected desmoid tumors, particularly in patients with a history of prior abdominal surgery, which may contribute to desmoid tumor development through postoperative remodeling or inflammatory changes. Although desmoid tumors typically present as solitary lesions, multifocal involvement can occur, particularly in sporadic cases [8].

Previous reports have shown that most intra-abdominal desmoid-type fibromatoses originate from the mesentery and are commonly associated with FAP [2]. In contrast, our case involved a sporadic tumor arising directly from the muscularis propria of the jejunum, which is extremely rare. While solitary lesions confined to the bowel wall have been reported [6], intraoperative identification of multiple additional lesions in the mesentery, as seen in our case, is uncommon.

To avoid short bowel syndrome while achieving curative resection, we extensively mobilized the duodenum and performed the FEEA near the ligament of Treitz. This approach allowed for maximal preservation of bowel length and minimized the risk of complications such as anastomotic leakage.

Desmoid tumors recur in 25%-50% of cases [9], necessitating long-term surveillance. Although the Ki-67 index in this case was low (2%), an intensive follow-up schedule was adopted due to the multifocal nature of the disease and the uncertain biological behavior of sporadic intestinal wall desmoid tumors. Follow-up with contrast-enhanced CT was scheduled every three months for the first two years and every six months thereafter until five years postoperatively. This strategy reflects institutional caution and aims at early detection of potential recurrence.

Recent European guidelines emphasize personalized and multidisciplinary treatment strategies [10,11]. However, the optimal management of desmoid tumors of the intestinal wall remains unclear. Our case highlights several key issues including diagnostic uncertainty, multifocality, and surgical planning. Accumulation of similar cases and long-term outcomes will contribute to the establishment of a comprehensive strategy for managing intestinal desmoid tumors.

## Conclusions

This case highlights the diagnostic and therapeutic challenges associated with solitary desmoid-type fibromatosis arising from the small intestine in the absence of familial adenomatous polyposis. The tumor’s submucosal origin, absence of mucosal involvement, and multifocal presentation complicated preoperative diagnosis and required careful intraoperative evaluation. Complete resection with adequate margins and functional reconstruction allowed for curative intent while preserving intestinal length. Given the tumor’s potential for local invasion and recurrence, long-term follow-up is essential, ideally for at least five years, as desmoid tumors have a known tendency for late recurrence. In this case, follow-up has been conducted for three months to date, and further surveillance is planned. This report contributes to the growing body of literature on rare, sporadic intestinal desmoid tumors and provides valuable insights into their surgical management.
